# Neoadjuvant Chemotherapy Does Not Improve Survival in cT2N0M0 Gastric Adenocarcinoma Patients: A Multicenter Propensity Score Analysis

**DOI:** 10.1245/s10434-024-15418-2

**Published:** 2024-05-18

**Authors:** Francesco Abboretti, Céline Lambert, Markus Schäfer, Bruno Pereira, Bertrand Le Roy, Diane Mège, Guillaume Piessen, Johan Gagnière, Caroline Gronnier, Styliani Mantziari, the ADENOKGAST Study Group, the AFC-French Association of Surgery

**Affiliations:** 1 Department of Visceral Surgery, Lausanne University Hospital, CHUV, Lausanne, Switzerland; 2 Faculty of Biology and Medicine, University of Lausanne UNIL, Lausanne, Switzerland; 3 Biostatistics Unit, DRCI, CHU Clermont-Ferrand, Clermont-Ferrand, France; 4 Department of Digestive and Oncologic Surgery, Hospital Nord, CHU de Saint-Etienne, Saint-Priest-en-Jarez, France; 5 Department of Digestive Surgery, Aix Marseille Univ, APHM, Timone University Hospital, Marseille, France; 6 Department of Digestive and Oncological Surgery, CHU Lille, Lille, France; 7 Univ. Lille, CNRS, Inserm, CHU Lille, UMR9020-U1277 - CANTHER – Cancer Heterogeneity Plasticity and Resistance to Therapies, Lille, France; 8 Department of Digestive Surgery and Liver Transplantation, Estaing University Hospital, Clermont-Ferrand, France; 9 U1071 Inserm/Clermont-Auvergne University, Clermont-Ferrand, France; 10 Eso-Gastric Surgery Unit, Department of Digestive Surgery, Magellan Center, Bordeaux University Hospital, Pessac, France; 11 Faculty of Medicine, Bordeaux Ségalen University, Bordeaux, France

**Keywords:** Stomach neoplasms, Neoadjuvant therapy, Survival analysis, Propensity score

## Abstract

**Background:**

According to current international guidelines, stage cT2N0M0 gastric adenocarcinoma warrants preoperative chemotherapy followed by surgery. However, upfront surgery is often preferred in clinical practice, depending on patient clinical status and local treatment preferences.

**Objective:**

The aim of the present study was to assess the impact of neoadjuvant chemotherapy in overall survival (OS) and disease-free survival (DFS) of cT2N0M0 patients.

**Methods:**

A retrospective analysis was performed among 32 centers, including gastric adenocarcinoma patients operated between January 2007 and December 2017. Patients with cT2N0M0 stage were divided into upfront surgery (S) and neoadjuvant chemotherapy followed by surgery (CS) groups. Inverse probability of treatment weighting (IPTW) was used to compensate for baseline differences between the groups.

**Results:**

Among the 202 patients diagnosed with cT2N0M0 stage, 68 (33.7%) were in the CS group and 134 (66.3%) were in the S group. CS patients were younger (mean age 62.7 ± 12.8 vs. 69.8 ± 12.1 years for S patients; *p *< 0.001) and had a better health status (World Health Organization performance status = 0 in 60.3% of CS patients vs. 34.5% of S patients; *p* = 0.006). During follow-up, recurrence occurred in 27.2% and 19.6% of CS and S patients, respectively, after IPTW (*p *= 0.32). Five-year OS was similar between CS and S patients (78.9% vs. 68.3%; *p *= 0.42), as was 5-year DFS (70.4% vs. 68.5%; *p *= 0.96). Neoadjuvant chemotherapy was associated with neither OS nor DFS in multivariable analysis after IPTW.

**Conclusions:**

Patients with cT2N0M0 gastric adenocarcinoma did not present a survival or recurrence benefit if treated with perioperative chemotherapy followed by surgery as opposed to surgery alone.

**Supplementary Information:**

The online version contains supplementary material available at 10.1245/s10434-024-15418-2.

Gastric and gastroesophageal junction cancer represent a major cause of cancer-related deaths worldwide.^1^ In locally advanced non-metastatic gastroesophageal adenocarcinoma, neoadjuvant chemotherapy offers a significant benefit in overall survival (OS) and disease-free survival (DFS) survival.^2,3^ In Europe and North America, perioperative chemotherapy is routinely performed for locally advanced disease (defined as ≥T2, *N* ≠ 0),^4^ with the aim to obtain tumor downstaging and reduce the risk of distant metastasis.^2,5^ However, despite the European Society for Medical Oncology (ESMO) guidelines^6^ suggesting chemotherapy for cT2N0 patients, clinical practice remains controversial as to whether this stage should be considered as early-stage or locally advanced. Of note, the MAGIC trial included only patients with tumor stage >Ib (T2N0).^2^ In the more recent FLOT-4 trial, subgroup analysis showed a benefit from neoadjuvant FLOT compared with ECF/ECX in cT2 tumors, although it remains unclear how many patients had positive lymph nodes during histological analysis.^3^ Similar studies from esophageal cancer^7^ have established that upfront surgery should be recommended for cT2N0 patients, as no survival benefit was observed for neoadjuvant treatment.

According to American (National Comprehensive Cancer Network [NCCN]) and European (ESMO) guidelines,^4,8^ neoadjuvant chemotherapy may be proposed to patients with T2N0 disease, but the strength of recommendation for this stage is low. A recent study by Gabriel et al.^9^ found no survival differences for ‘true’ T2N0 patients (cT2N0/pT2N0) treated with neoadjuvant chemotherapy followed by surgery, versus surgery alone. However, ‘true’ T2N0 stage is difficult to ascertain, as despite recent advances in the accuracy of diagnostic methods, staging errors remain frequent in gastric cancer patients, especially in the diffuse type.^10^ Thus, informed decision making in multidisciplinary tumor boards mandates further evidence to support whether systemic chemotherapy is needed in cT2N0 patients.

The aim of this multicenter cohort study was to assess long-term survival and recurrence in patients with cT2N0 gastric adenocarcinoma treated with chemotherapy followed by surgery, versus upfront surgery.

## Methods

### Inclusion Criteria

All consecutive adult patients (>18 years of age) who underwent surgery with curative intent for gastric or esophagogastric junction (Siewert III) adenocarcinoma between January 2007 and December 2017 in the 32 participating centers (Online Resource 1) were assessed for inclusion. Exclusion criteria were histological type other than adenocarcinoma (e.g. gastrointestinal stromal tumor, neuroendocrine tumor, lymphoma) or documented patient refusal to participate in clinical research. Patients with clinical T2N0M0 stage at initial diagnosis were identified and included in the current study.

### Study Endpoints

The primary endpoint was to assess the impact of neoadjuvant chemotherapy in OS and DFS in the cT2N0M0 patients. Postoperative (in-hospital) morbidity and mortality, as well as recurrence patterns, were assessed as secondary endpoints. For all analyses, cT2N0M0 patients were divided into two groups: chemotherapy followed by surgery (CS group) or upfront surgery (S group). Of note, DFS was calculated after excluding patients who experienced postoperative mortality and patients with metastatic disease upon diagnosis.

### Data Collection, Treatment and Follow-Up

Baseline demographic, histological, and treatment data were collected from all eligible patients. Surgical resection and perioperative treatment were performed in all centers, based on the ESMO guidelines.^6^ Lymph node dissection was commonly defined as D1.5 or spleen-preserving D2. Chemotherapy regimens varied across centers and local practice, with most common combinations being the ECF/ECX^2^ and, more recently, FLOT regimens.^3^ Postoperative complications were assessed during the entire hospital stay of the index operation and graded according to the validated 5-scale Dindo–Clavien classification, with grades >IIIa defined as ‘major’.^11^ Tumor regression grade (TRG) was evaluated by pathologists according to Mandard score.^12^ Long-term follow-up was performed by thoraco-abdominal computed tomography (CT) scan and/or upper endoscopy on demand,^13^ according to local protocols. When tumor recurrence was detected, it was classified as locoregional (peri-anastomotic, locoregional lymph nodes), distant (solid organ metastasis, peritoneal carcinomatosis), or mixed. The median follow-up in the current series was 32.3 months (interquartile range [IQR] 20.1–62.3) for the S group and 30.2 months (IQR 17.9–52.6) for the CS group.

### Statistical Analysis

Statistical analyses were performed using Stata software (version 15; StataCorp LLC, College Station, TX, USA). All tests were two-sided, with an alpha level set at 5%. Categorical data are presented as the number of patients and associated percentages, and continuous data as mean ± standard deviation. Comparisons between the independent groups (S and CS) were performed using the Chi-square test or Fisher’s exact test for categorical variables, and the Student’s t test or Mann–Whitney test for continuous variables. To compensate for baseline differences between the groups, a propensity score (PS) analysis was implemented using the inverse probability of treatment weighting (IPTW) method,^14,15^ which consists of creating a ‘pseudo sample’ of treated (CS) and untreated (S) patients, weighting each patient by the inverse probability of receiving the treatment he or she actually received: 1/PS in the CS group and 1/(1-PS) in the S group. In practice, the probability of receiving chemotherapy before surgery was modeled using multiple logistic regression, and the estimated probability was used as the PS. Baseline variables that might have affected treatment decisions were selected for the PS based on clinical relevance, i.e. age, World Health Organization (WHO) performance status, and tumor location. Balance between groups was measured by standardized mean differences (expressed as absolute values), and a value >0.2 was considered a sign of imbalance. Censored data (OS and DFS) were estimated using the Kaplan–Meier method, and the groups were compared using the Cox model, considering the center as a random effect. Factors associated with OS and DFS at 5 years were also studied using the Cox model, and the results are expressed as hazard ratio (HR) and 95% confidence interval (CI).

### Ethical Considerations

The current study was approved by the respective Ethics Committees of the participating centers (ADENOKGAST protocol, Clermont-Ferrand University Hospital, France [IRB 00013412, 2022-CF030], and Vaud Ethics Committee in Switzerland [CER-VD ID Number 2022-02262]). Each center was responsible for approval by the local Institutional Review Board.

## Results

Overall, 2131 patients were included in the multicenter gastric adenocarcinoma (ADENOKGAST) cohort, across 32 participating French-speaking centers (Online Resource 1). A clinical T2N0M0 stage was diagnosed in 202 patients (9.5%), among whom 134 patients (66.3%) underwent upfront surgery (S group), while 68 patients (33.7%) received neoadjuvant chemotherapy followed by surgery (CS group).

### Clinicopathological and Surgical Characteristics

Baseline patient and treatment characteristics are presented in Table 1. Before IPTW, the mean age was 69.8 ± 12.1 years in the S group, versus 62.7 ± 12.8 in the CS group (*p* < 0.001). CS patients had a better health status (WHO performance status = 0 in 60.3% of CS patients vs. 34.5% of S patients; *p* = 0.006). More proximal tumors (cardia, gastric fundus) were found in the CS group (61.5%, vs. 42.6% in the S group; *p* = 0.001), and total gastrectomy rates differed accordingly (70.6% in the CS group vs. 45.5% in the S group; *p* = 0.001). Patients in the CS group received adjuvant chemotherapy more often compared with the S group (75.8% vs. 29.8%; *p* < 0.001). All clinicopathological and surgical variables were comparable between CS and S patients after IPTW (Table 1).

**Table 1:** Demographic and surgical characteristics, and postoperative morbidity of patients with cT2N0 gastric cancer receiving upfront surgery (S) versus neoadjuvant chemotherapy plus surgery (CS) before and after applying inverse probability weighting

Variable	Before inverse probability of treatment weighting				After inverse probability of treatment weighting				SMD
	All [*N* = 202]	S [*n* = 134]	CS [*n* = 68]	*p*-Value	All	S	CS	*p*-Value	
Male sex	119/201 (59.2)	79 (59.0)	40/67 (59.7)	0.92	(58.9)	(59.3)	(58.5)	0.93	0.02
Age, years	67.3 ± 12.8	69.8 ± 12.1	62.7 ± 12.8	**<0.001**	65.8 ± 13.0	65.8 ± 14.1	65.9 ± 11.8	0.96	0.01
WHO performance status				**0.006**				0.95	
0	78/179 (43.6)	40/116 (34.5)	38/63 (60.3)		(49.1)	(50.2)	(48.0)		0.04
1	81/179 (45.3)	59/116 (50.9)	22/63 (34.9)		(43.6)	(43.2)	(44.0)		0.02
2	16/179 (8.9)	13/116 (11.2)	3/63 (4.8)		(7.3)	(6.6)	(8.0)		0.05
3	4/179 (2.2)	4/116 (3.4)	0/63 (0.0)		(0.0)	(0.0)	(0.0)		NA
Smoking	65/172 (37.8)	43/113 (38.1)	22/59 (37.3)	0.92	(40.8)	(46.6)	(34.7)	0.19	0.24
Alcohol consumption	29/163 (17.8)	23/106 (21.7)	6/57 (10.5)	0.08	(20.8)	(24.8)	(16.9)	0.40	0.20
Tumor location				**0.001**				0.96	
Proximal	95/194 (49.0)	55/129 (42.6)	40/65 (61.5)		(55.1)	(56.3)	(53.8)		0.05
Body	15/194 (7.7)	15/129 (11.6)	0/65 (0.0)		(0.0)	(0.0)	(0.0)		NA
Distal	77/194 (39.7)	56/129 (43.4)	21/65 (32.3)		(42.2)	(41.0)	(43.5)		0.05
Diffuse	7/194 (3.6)	3/129 (2.3)	4/65 (6.2)		(2.7)	(2.7)	(2.7)		0.00
Surgery approach				0.47				0.13	
LS	15/201 (7.5)	12/133 (9.0)	3 (4.4)		(5.1)	(7.9)	(2.2)		0.26
LS converted to LT	2/201 (1.0)	2/133 (1.5)	0 (0.0)		(1.2)	(2.3)	(0.0)		0.22
LT	183/201 (91.0)	118/133 (88.7)	65 (95.6)		(92.9)	(88.2)	(97.8)		0.38
TS converted to TT	1/201 (0.5)	1/133 (0.8)	0 (0.0)		(0.8)	(1.6)	(0.0)		0.18
Lymph node dissection				0.56				0.82	
D1	28/195 (14.4)	21/129 (16.3)	7/66 (10.6)		(15.7)	(17.8)	(13.5)		0.12
D1.5	124/195 (63.6)	80/129 (62.0)	44/66 (66.7)		(63.9)	(61.6)	(66.3)		0.10
D2	43/195 (22.0)	28/129 (21.7)	15/66 (22.7)		(20.4)	(20.6)	(20.2)		0.01
Total gastrectomy	109 (54.0)	61 (45.5)	48 (70.6)	**0.001**	(58.6)	(50.3)	(67.0)	0.07	0.34
Dindo–Clavien grade				0.98				0.15	
I/II/IIIa	123/163 (75.5)	80/107 (74.8)	43/56 (76.8)		(81.2)	(84.1)	(78.2)		0.15
IIIb/Iva/IVb	24/163 (14.7)	16/107 (15.0)	8/56 (14.3)		(12.6)	(13.5)	(11.6)		0.06
V	2/163 (1.2)	1/107 (0.9)	1/56 (1.8)		(0.5)	(0.9)	(0.0)		0.14
Other	14/163 (8.6)	10/107 (9.3)	4/56 (7.1)		(5.7)	(1.5)	(10.2)		0.38
Surgical complications	53 (26.2)	35 (26.1)	18 (26.5)	0.96	(23.3)	(30.4)	(16.0)	**0.049**	0.35
Re-intervention	25/184 (13.6)	16/120 (13.3)	9/64 (14.1)	0.89	(13.0)	(12.7)	(13.2)	0.93	0.02
Medical complications	56 (27.7)	40 (29.9)	16 (23.5)	0.34	(27.7)	(31.5)	(23.7)	0.38	0.17
Adjuvant chemotherapy	89/197 (45.2)	39/131 (29.8)	50/66 (75.8)	**<0.001**	(56.8)	(38.5)	(75.3)	**<0.001**	0.80

Bold values denote statistical significance at the *p* < 0.05 level

Data are expressed as number of patients (percentages) or mean ± standard deviation

*LS* laparoscopy, *LT* laparotomy, *NA* not applicable, *SMD* standardized mean difference (in absolute value), *TS* thoracoscopy, *TT* thoracotomy, *WHO* World Health Organization

Histopathological analysis revealed no differences in R0 resection rates (95.6% in the CS group vs. 95.5% in the S group; *p* = 1.00) [Table 2]. Stage pT1-pT2 lesions were more frequently observed in the S group (60.9%, vs. 50.7% in the CS group), while pT0-pTis lesions were more frequent in the CS group (8.9%, vs. 0.8% in the S group; *p* = 0.01). There was no difference in histological subtype.

**Table 2:** Histopathological characteristics of patients with cT2N0 gastric cancer receiving upfront surgery (S) versus neoadjuvant chemotherapy plus surgery (CS) before and after applying inverse probability weighting

Variable	Before inverse probability of treatment weighting				After inverse probability of treatment weighting				SMD
	All [*N* = 202]	S [*n* = 134]	CS [*n* = 68]	*p*-Value	All	S	CS	*p*-Value	
Tumor differenciation				0.38				0.89	
G1	35/148 (23.7)	26/96 (27.1)	9/52 (17.3)		(18.3)	(20.8)	(15.9)		0.13
G2	59/148 (39.9)	39/96 (40.6)	20/52 (38.5)		(44.0)	(44.3)	(43.8)		0.01
G3	52/148 (35.1)	30/96 (31.3)	22/52 (42.3)		(36.1)	(33.7)	(38.4)		0.10
G4	2/148 (1.4)	1/96 (1.0)	1/52 (1.9)		(1.6)	(1.2)	(1.9)		0.05
Resection margins				1.00				0.92	
R0	193 (95.5)	128 (95.5)	65 (95.6)		(95.5)	(95.7)	(95.3)		0.02
R1	9 (4.5)	6 (4.5)	3 (4.4)		(4.5)	(4.3)	(4.7)		0.02
pT stage				**0.01**				0.08	
pT0–pTis	7/200 (3.5)	1/133 (0.8)	6/67 (8.9)		(4.2)	(0.0)	(8.5)		0.43
pT1–pT2	115/200 (57.5)	81/133 (60.9)	34/67 (50.7)		(55.0)	(55.5)	(54.5)		0.02
pT3–pT4	78/200 (39.0)	51/133 (38.4)	27/67 (39.7)		(40.8)	(44.5)	(36.9)		0.16
pN stage				0.12				**0.005**	
pN0	121/198 (61.1)	75/132 (56.8)	46/66 (69.7)		(61.6)	(51.0)	(72.5)		0.45
pN1	42/198 (21.2)	28/132 (20.2)	14/66 (20.2)		(20.4)	(19.7)	(21.1)		0.03
pN2	22/198 (11.1)	19/132 (14.4)	3/66 (4.6)		(11.2)	(18.0)	(4.3)		0.45
pN3	13/198 (6.6)	10/132 (7.6)	3/66 (4.6)		(6.8)	(11.3)	(2.1)		0.37
WHO tumor classification				0.37				0.77	
Signet-ring cell	57/155 (36.8)	38/107 (35.5)	19/48 (39.6)		(40.4)	(43.6)	(36.9)		0.14
Undifferentiated	4/155 (2.6)	1/107 (0.9)	3/48 (6.3)		(3.9)	(3.1)	(4.8)		0.08
Mucinous	12/155 (7.7)	7/107 (6.5)	5/48 (10.4)		(7.3)	(4.7)	(10.1)		0.21
Papillary	9/155 (5.8)	7/107 (6.5)	2/48 (4.2)		(2.6)	(2.0)	(3.4)		0.09
Tubular	73/155 (47.1)	54/107 (50.5)	19/48 (39.6)		(45.8)	(46.6)	(44.8)		0.04
Lauren tumor classification				0.63				**0.03**	
Diffuse	29/130 (22.3)	20/89 (22.5)	9/41 (22.0)		(19.6)	(24.8)	(12.8)		0.31
Intestinal	76/130 (58.5)	50/89 (56.2)	26/41 (63.4)		(60.8)	(49.9)	(75.2)		0.54
Mixed	25/130 (19.2)	19/89 (21.4)	6/41 (14.6)		(19.6)	(25.3)	(12.0)		0.35
Poorly cohesive histology	67/183 (36.6)	43/120 (35.8)	24/63 (38.1)	0.76	(38.4)	(41.5)	(35.2)	0.49	0.13

Bold values denote statistical significance at the *p* < 0.05 level

Data are expressed as number of patients (percentages)

*SMD* standardized mean difference (in absolute value), *WHO* World Health Organization

### Neoadjuvant Chemotherapy Details and Toxicity Profile

Within the CS group, the most used regimen was ECF (epirubicin, cisplatin, and 5-fluorouracil) (91.2%, *n* = 62), with only three patients (4.4%) receiving the more recent FLOT (5-fluorouracil, leucovorin, oxaliplatin, docetaxel). Severe chemotherapy-related toxicity (grade ≥3) was observed in 7.4% of CS patients. Upon histopathologic analysis, 15 patients in the CS group presented a good response to chemotherapy (TRG 1–2), accounting for 60% of CS patients for whom this variable was available (*n* = 25/68). After neoadjuvant chemotherapy (*n* = 68), restaging radiologic work-up revealed disease progression in 1.5% of patients, stable disease in 32.4%, partial response in 26.5%, and complete response in 4.4% of patients.

### Postoperative Morbidity

As illustrated in Table 1, minor postoperative complications occurred in 74.8% of the S group vs. 76.8% of the CS group, whereas major complications (Dindo–Clavien >IIIa) occurred in 15.0% and 14.3%, respectively (*p* = 0.98). The re-intervention rate was also similar (13.3% for the S group vs. 14.1% for the CS group; *p* = 0.89). No differences were seen in postoperative (in-hospital) mortality (0.9% for the S group vs. 1.8% for the CS group). After applying IPTW, surgical complications were more prevalent in the S group (30.4%) compared with 16.0% in the CS group (*p* = 0.049).

### Long-Term Survival and Recurrence

**Fig. 1:**
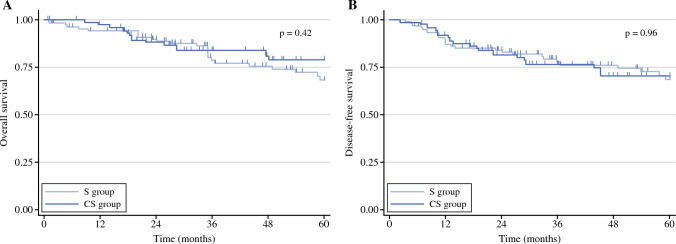
Five-year (**A**) overall survival and (**B**) disease-free survival of patients with cT2N0 gastric cancer receiving upfront surgery (S) versus neoadjuvant chemotherapy plus surgery (CS), after inverse probability of treatment weighting. *S group* upfront surgery, *CS group* neoadjuvant chemotherapy plus surgery

**Fig. 2:**
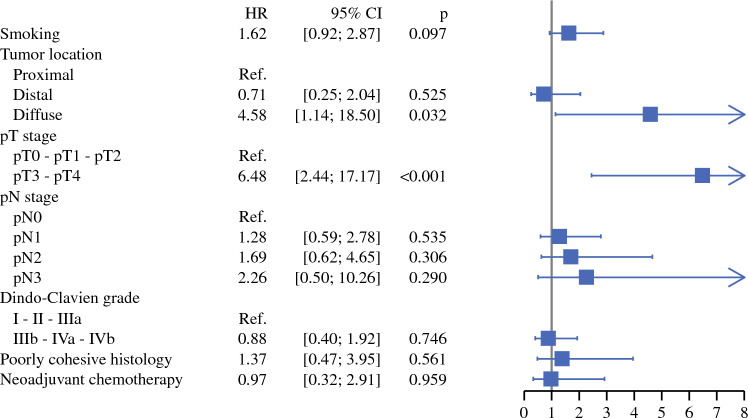
Multivariable analysis of factors associated with a worse overall survival at 5 years after inverse probability of treatment weighting. *CI* confidence interval, *HR* hazard ratio

After IPTW, 5-year OS was similar between the S and CS groups—68.3% and 78.9%, respectively (*p* = 0.42) [Fig. 1]. Similarly, 5-year DFS was 68.5% in the S group vs. 70.4% in the CS group (*p* = 0.96) [Fig. 1]. Multivariable Cox regression revealed no impact of neoadjuvant chemotherapy on OS after IPTW (adjusted HR 0.97, 95% CI 0.32–2.91; *p* = 0.96). Diffuse histology (adjusted HR 4.58, 95% CI 1.14–18.5; *p* = 0.032) and pT3-pT4 stage (adjusted HR 6.48, 95% CI 2.44–17.17; *p* < 0.001) were independent predictors of poor OS (Fig. 2). Detailed results of univariate and multivariable Cox regression for OS are presented in Online Resource 2.

Overall recurrence rate was higher in the CS group during follow-up (30.9%, vs. 18.0% in the S group; *p* = 0.04), without differences in recurrence patterns (locoregional, 12.5% in the S group vs. 14.3% in the CS group; distant metastatic, 87.5% in the S group vs. 85.7% in the CS group; *p* = 1.00). After IPTW, no differences were found in the overall recurrence rate (19.6% in the S group vs. 27.2% in the CS group; *p* = 0.32) nor in the rate of metastatic recurrence site (90.6% in the S group vs. 83.5% in the CS group; *p* = 0.53).

**Fig. 3:**
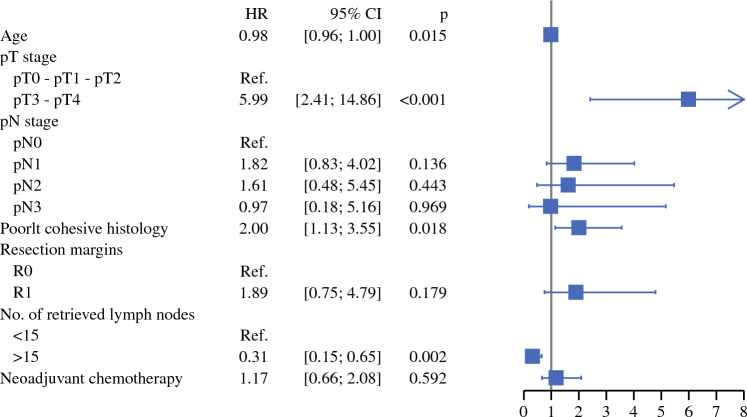
Multivariable analysis of factors associated with a worse disease-free survival at 5 years after inverse probability of treatment weighting. *CI* confidence interval, *HR* hazard ratio

As illustrated in Fig. 3, neoadjuvant chemotherapy was not significantly associated with DFS in multivariable analysis after IPTW (adjusted HR 1.17, 95% CI 0.66–2.08; *p* = 0.59). Pathological pT3-pT4 stage (adjusted HR 5.99, 95% CI 2.41–14.86; *p* < 0.001) and signet ring cell histology (adjusted HR 2.00, 95% CI 1.13–3.55; *p* = 0.02) were independently associated with poor DFS, whereas younger age (adjusted HR 0.98, 95% CI 0.96–1.00; *p* = 0.015) and lymphadenectomy with ≥15 retrieved lymph nodes was associated with more favorable DFS (adjusted HR 0.31, 95% CI 0.15–0.65; *p* = 0.002). Detailed results of univariate and multivariable Cox regression for DFS are presented in Online Resource 3.

## Discussion

This European multicenter cohort study evaluated postoperative and long-term outcomes in patients with cT2N0M0 gastric adenocarcinoma who underwent either upfront surgery or neoadjuvant chemotherapy followed by surgery. Neoadjuvant chemotherapy did not increase postoperative morbidity, and neither did it offer a benefit in OS or decreased recurrence rate in cT2N0 stage patients.

The subgroup of cT2N0 stage patients remains underrepresented in the scientific literature. In a recent retrospective study, Gabriel et al. suggested the absence of survival benefit in cT2N0 patients after neoadjuvant chemotherapy compared with upfront surgery for patients with a ‘true’ T2N0 status (both cT2N0 and pT2N0).^9^ In line with these findings, in our series, OS and DFS were also similar for cT2N0 patients, irrespective of neoadjuvant chemotherapy administration. On a multivariable level, signet-cell histology and locally advanced cT stage were associated with poor OS. Advanced cT stage, signet ring cell histology, and limited lymphadenectomy (a <15 lymph node yield) were associated with earlier recurrence.

The present findings are in contrast with the current European (ESMO)^6^ and North American (NCCN) gastric cancer treatment guidelines,^8^ which suggest neoadjuvant chemotherapy for cT2N0 disease. Interestingly, the current series conducted in 32 European reference centers reveal that 66.3% of cT2N0 patients underwent upfront surgery. How might this divergence from treatment guidelines be explained? Our results illustrate that chemotherapy is more frequently offered to younger patients, with a better overall health status and more proximal tumors. Although the individual motivation behind treatment choices cannot be retrieved for all patients, a tendency to ‘spare’ chemotherapy to more frail patients with distal tumors has been observed. As previously published by Handforth et al., ‘unfit’ but operable oncological patients could still benefit from surgical resection, as fragile individuals are more likely to experience chemotherapy intolerance, surgical complications, and death.^16^ This is in accordance with previous reports on esophageal cancer patients, where acceptable oncological outcomes were reported when ‘chemotherapy-unfit patients’ underwent upfront surgery for early disease stage.^17^ Interestingly, in the present study, a higher rate of complications was observed in the S group, despite the lower rate of total gastrectomy. Although specific data to explain this difference are not available in our study, we can hypothesize that patient frailty, more pronounced in this group as discussed above, might have a role in increasing surgical morbidity.

In recent years, the FLOT chemotherapy regimen has prevailed in gastric cancer treatment, with superior efficacy compared with the MAGIC (ECF) protocol.^2^ As, in the present study, most patients were treated in the pre-FLOT era, the efficacy of this regimen for T2N0 disease cannot be assessed, and improved patient outcomes may be observed in the future. However, it needs to be kept in mind that the increased efficacy of the FLOT regimen often comes with the price of increased toxicity rates. In our series, with predominantly ECF-based chemotherapy, severe toxicity was documented in 7.4% of patients, which is in the lower range of similar reports; in the FNCLCC trial, grade III–IV toxicity after ECF chemotherapy reached 38%,^5^ whereas previous studies report rates of 26–50%.^18,19^ Al-Batran et al. observed a 27% grade III toxicity rate in the original FLOT trial.^3^ Thus, the increased risk of severe toxicity needs consideration in the FLOT era, and indications to this treatment need to be weighed accordingly.

Interestingly, a higher incidence of proximal tumors was seen in the CS group, resulting in higher rates of total gastrectomy compared with patients who underwent upfront surgery. Although a clear explanation for this difference in tumor epicenter cannot be provided, some potential biomolecular implications may be hypothesized. The Cancer Genomic Atlas (TCGA) Project identified four distinct molecular subtypes of gastric cancer, with proximal tumors being often chromosomally unstable, with a high incidence of TP53 mutation and RTK-RAS activation.^20^ Even if the TCGA molecular subtyping of gastric cancer has no direct clinical implications yet, it has been reported that patients with proximal tumors experience poorer survival compared with patients with distal tumors.^21–24^ A retrospective study from 2018 suggested that proximal tumors are often associated with more advanced disease presentation and poor prognosis,^25^ whereas another study found no survival differences between proximal and distal tumors.^26^ Wang et al. found a poor prognosis for patients with proximal tumors in both early and locally advanced stage, while in a metastatic stage, the prognosis of distal tumors was worse.^27^ It could be hypothesized that the unfavorable biomolecular substrate of proximal gastric cancer might warrant systemic chemotherapy in this group of patients, even in early-stage (cT2N0) disease.

The issue of poor diagnostic accuracy in the preoperative staging of gastric cancer needs to be emphasized. Even with current diagnostic methods, understaging was observed in 38% of patients in our series. A high occurrence of pT3-pT4 disease was found in both the S and CS groups (38.4% and 39.7%, respectively), whereas extensive lymph node invasion was also frequent upon final pathology (pN3 in 7.6% of S patients and 4.6% of CS patients). Our results concord with those published by Ju et al., where almost 40% of patients with cT1-2N0M0 gastric adenocarcinoma were understaged, mostly often underestimating lymph node extension.^28^ In the current study, only 56.8% of cT2N0 patients in the S group were actually pN0 upon histological analysis, which is consistent with the respective rates (60%) reported in esophageal cancer.^29^ The use of endoscopic ultrasound in the preoperative staging of gastric cancer has significantly improved staging accuracy; however, it also has limitations, being operator-dependent and with poorer discriminatory capacity in case of diffuse-type cancer and assessment of distant lymph nodes.^30,31^ After IPTW, our data indicate significant differences in pN stage between the groups, particularly a higher proportion of pN0 stage and significantly lower proportions of pN2 and pN3 stages in the CS group compared with the S group. This suggests that neoadjuvant chemotherapy may reduce the risk of microscopic lymphatic dissemination in cT2N0 gastric cancer patients. Therefore, the decision to refrain from systemic chemotherapy in a fit patient, especially with a proximal gastric tumor, needs to be carefully considered, as the risk of underestimating the baseline disease stage is considerable and where the potential benefits of reducing lymph node tumor burden might be more pronounced. The observed similarity in 5-year DFS between groups, despite higher overall recurrence in the CS group, may be explained by the DFS metric accounting for both recurrences and deaths as events, and its consideration of the timing of these events. Notably, post-IPTW analysis, which adjusts for baseline differences, shows no significant difference in overall recurrence, suggesting that initial disparities may be attributed to imbalances in patient characteristics prior to weighting.

Lastly, in the current study, a higher rate of CS patients were found to receive postoperative chemotherapy. Although the reasons behind individual treatment choices cannot be explained retrospectively, in many participating centers adjuvant chemotherapy is most often reserved for patients previously selected for neoadjuvant chemotherapy. This is especially true if patient frailty had driven the preoperative decision, as it is known that adjuvant chemotherapy may be tolerated even less well than neoadjuvant therapy.^6^

The current study presents several limitations that need to be discussed. First, the sample size of the cT2N0M0 group is rather limited due to the relative rarity of this stage in the patient population (9.5% in the entire cohort). However, this study represents one of the largest European cohorts specifically examining cT2N0M0 patients, thus offering conclusions applicable to clinical practice. A further significant limitation of this study is its retrospective design with the inherent problem of missing data, notably for specific biomolecular markers such as microsatellite stability/microsatellite instability (MSS/MSI) status, which might influence and explain tumor biology and response to chemotherapy. Similarly, our database lacks data on patients who initiated chemotherapy but were unable to proceed to surgery due to treatment-related toxicity. Some significant baseline differences were observed between the two groups (S and CS). A PS matching with IPTW was performed to match for the main confounders, and multivariable analyses were also performed for the primary outcomes of interest. However, other unknown confounders may also be present and influence the current results. In addition, the ECF (MAGIC) protocol predominantly used in our study has nowadays largely been replaced by the FLOT protocol, potentially influencing the applicability of our findings to current clinical practice. While acknowledging this drawback, we do believe that the current analysis of the previous gold-standard ECF treatment does not lack clinical validity. First, the ECF regimen has proven efficient for decades in gastric cancer patients, and thus not all retrospective series using this regimen can be considered unreliable. Second, although the FLOT regimen is currently dominating treatment standards in many centers, its actual rate of use in everyday practice remains unknown, as it is associated with significant toxicity often mandating alternative and less toxic regimens (e.g. FLOT, ECF).

Finally, one might argue that the staging discrepancies observed (cT2N0 ≠ pT2N0) might limit the validity of our findings for ‘true’ T2N0 disease. However, our analysis is focused on clinically staged T2N0 patients, with the specific purpose to provide helpful evidence during initial decision making and guide multidisciplinary tumor board discussions. Thus, although separate analysis of the ‘real’ cT2N0/pT2N0 patients was not opted for, multivariable analysis was performed, showing no independent prognostic value for the pN stage.

## Conclusion

Our results suggest that neoadjuvant chemotherapy, predominantly represented by the ECF regimen in this series, had no significant impact on short- and long-term outcomes in patients with cT2N0M0 gastric adenocarcinoma. Our results suggest that upfront surgery could be a viable treatment option in this group, as in esophageal cT2N0 stage,^7^ especially in frail patients with distally located lesions. Further research is needed to identify specific molecular phenotypes predisposing to aggressive disease course in order to identify potential subgroups of cT2N0 patients who could benefit from systemic treatment.

### Supplementary Information

Below is the link to the electronic supplementary material.

Supplementary file1 (DOCX 15 KB)

Supplementary file2 (DOCX 22 KB)

Supplementary file3 (DOCX 20 KB)
