# Supplementary material for: Neoadjuvant Chemotherapy Does Not Improve Survival in cT2N0M0 Gastric Adenocarcinoma Patients: A Multicenter Propensity Score Analysis
Source: Ann Surg Oncol. 2024 May 18;31(8):5273–82. doi: 10.1245/s10434-024-15418-2 (PMC11236876; doi:10.1245/s10434-024-15418-2)
Supplement: Supplementary file 1 — Supplementary file1 (DOCX 15 KB) [file 10434_2024_15418_MOESM1_ESM.docx]

**Online Resource 1.** ADENOKGAST authors list.

Amiens, CHU: Jean-Marc Regimbeau, Baptiste Brac, unité de recherche clinique – Angers CHU: Émilie Lermite, Julien Barbieux, Elodie Cloche, Pierre Gueroult – Aurillac, CH: Serban Puia-Negulsecu – Bordeaux, CHU: Caroline Gronnier, Olivier Degrandi, Camille Joumaa, Martin Seel, Damien Bouriez, Soline Celarier, Denis Collet – Brest, CHU: Jérémie Théréaux, Coralie Roche – Bruxelles, ULB Erasme- Bordet – Clermont-Ferrand, CHU: Denis Pezet, Julie Veziant, Johan Gagnière, Ophélie Bacœur-Ouzillou, Camille Lecomte, Flora Badon-Murgue. – Firminy, CH: Samer Al Mustafa – Le Puy-en-Velay, CH: Antoune Sarkis, Guy Lescure – Lille, CHRU: Anne Gandon, Clarisse Eveno, Georges Dubreuilh, Guillaume Piessen – Lille, clinique de La Louvière: Laurent Arnalsteen – Lyon, Hôpital Edouard-Herriot: Arnaud Pasquer, Antoine Breton, Maud Robert, Gilles Poncet – Marseille, la Timone, CHU: Diane Mège, Igor Sielezneff – Marseille, Institut Paoli Calmette: Jérome Guiramand, Olivier Turrini – Mont-de-Marsan, CH: Claude Chaussende, Victor Ewassadja, Rémy Chevalier – Montpellier, CHU:François-Régis Souche, Jean-Michel Fabre, Charlotte Ferrandis, Thomas Bardol, Lorrenzo Ferre – Paris, Hôpital de la Pitié-Salpétrière: Gilles Manceau, Mehdi Karoui – Paris, Hôpital Lariboisière: Marc Pocard, Dahbia Djelil – Paris, Hôpital Saint-Antoine: Thibault Voron, Jérémie H. Lefevre, Yann Parc, François Paye, Pierre Balladur – Paris, Institut Mutualiste Montsouris: David Fuks, Anne de Carbonnières, Stéphane Bonnet – Paris, Hôpital Saint-Louis: Pierre Cattan, Jonathan Demma, Hélène Corte – Paris, Hôpital Foch: Nicolas Margot, Alexandre Rault – Poitiers, CHU: Thomas Courvoisier-Clément, Jean-Pierre Faure – Rennes, CHU: Damien Bergeat, Edouard Wasielewski, Bernard Meunier – Saint-Étienne, CHU: Bertrand Le Roy, Don-André Vincentelli, Antoine Epin, Thomas Sole, Jérome Deheppe, Axel Denneval, Charlotte Pella – Saint-Flour: Dr Sergey Melnikov – Toulouse, CHU: Guillaume Péré, Nicolas Carrere – Vichy, CH: Florent Genty – Suisse, Lausanne, CHUV: Styliani Mantziari, Francesco Abboretti, Markus Schäfer, Nicolas Demartines – Italie, Milan Niguarda Hospital: Monica Gualtierotti – Burkina Faso: Adama Sanou, Rodrigue N Doamba, Roland O Somé – Maroc, Institut National Rabah Al Ahzar: Abdelilah Souadka, Amina Houmada – Maroc, Institut national d’oncologie Rabat: Amine Souadka, Sara El Atiq, Amine Benkabbou, Mohammed Anas Majbar, Raouf Mohsine – Algérie, Bejaia: Salah Berkane – Algérie, service de chirurgie A Tlemcen Dr Mesli
