# Supplementary material for: Neoadjuvant Chemotherapy Does Not Improve Survival in cT2N0M0 Gastric Adenocarcinoma Patients: A Multicenter Propensity Score Analysis
Source: Ann Surg Oncol. 2024 May 18;31(8):5273–82. doi: 10.1245/s10434-024-15418-2 (PMC11236876; doi:10.1245/s10434-024-15418-2)
Supplement: Supplementary file 2 — Supplementary file2 (DOCX 22 KB) [file 10434_2024_15418_MOESM2_ESM.docx]

**Online Resource 2.** Univariate and multivariable Cox regression analysis for overall survival at five years, after inverse probability of treatment weighting.

| **Variable** | **Unadjusted**  **HR** | **95%CI** | **P Value** |  | **Adjusted**  **HR** | **95%CI** | **P Value** |
| --- | --- | --- | --- | --- | --- | --- | --- |
| Male sex | 1.39 | 0.63; 3.08 | 0.41 |  |  |  |  |
| Age (years) | 0.98 | 0.95; 1.01 | 0.21 |  |  |  |  |
| WHO performance status |  |  |  |  |  |  |  |
| 0 | 1.00 |  |  |  |  |  |  |
| 1 | 1.29 | 0.63; 2.65 | 0.48 |  |  |  |  |
| 2 | 1.16 | 0.21; 6.35 | 0.87 |  |  |  |  |
| 3 | - | - | NA |  |  |  |  |
| Smoking | 1.74 | 0.99; 3.06 | 0.06 |  | 1.62 | 0.92; 2.87 | 0.10 |
| Alcohol consumption | 0.63 | 0.25; 1.55 | 0.31 |  |  |  |  |
| Tumor location |  |  |  |  |  |  |  |
| Proximal | 1.00 |  |  |  | 1.00 |  |  |
| Body | - | - | NA |  | - | - | - |
| Distal | 0.43 | 0.18; 1.03 | 0.06 |  | 0.71 | 0.25; 2.04 | 0.53 |
| Diffuse | 2.57 | 0.66; 10.03 | 0.17 |  | 4.58 | 1.14; 18.50 | **0.03** |
| pT stage |  |  |  |  |  |  |  |
| pT0 - pT1 - pT2 | 1.00 |  |  |  | 1.00 |  |  |
| pT3 - pT4 | 6.19 | 2.63; 14.56 | **<0.001** |  | 6.48 | 2.44; 17.17 | **<0.001** |
| pN stage |  |  |  |  |  |  |  |
| pN0 | 1.00 |  |  |  | 1.00 |  |  |
| pN1 | 3.13 | 1.55; 6.33 | **0.002** |  | 1.28 | 0.59; 2.78 | 0.54 |
| pN2 | 3.29 | 1.53; 7.09 | **0.002** |  | 1.69 | 0.62; 4.65 | 0.31 |
| pN3 | 3.86 | 1.15; 12.99 | **0.03** |  | 2.26 | 0.50; 10.26 | 0.29 |
| Dindo-Clavien grade |  |  |  |  |  |  |  |
| I - II - IIIa | 1.00 |  |  |  | 1.00 |  |  |
| IIIb - IVa - IVb | 1.88 | 1.01; 3.51 | **0.049** |  | 0.88 | 0.40; 1.92 | 0.75 |
| Neoadjuvant chemotherapy | 0.75 | 0.38; 1.50 | 0.42 |  | 0.97 | 0.32; 2.91 | 0.96 |
| Poorly cohesive histology | 2.11 | 0.94; 4.73 | 0.07 |  | 1.37 | 0.47; 3.95 | 0.56 |
| Resection margins |  |  |  |  |  |  |  |
| R0 | 1.00 |  |  |  |  |  |  |
| R1 | 2.15 | 0.42; 10.99 | 0.36 |  |  |  |  |
| Number of retrieved LN |  |  |  |  |  |  |  |
| <15 | 1.00 |  |  |  |  |  |  |
| ≥15 | 0.76 | 0.26; 2.20 | 0.61 |  |  |  |  |
| *CI: confidence interval; HR: hazard ratio; LN: lymph nodes; NA: not applicable; WHO: world health organization.* | | | | | | | |
