# Supplementary material for: Neoadjuvant Chemotherapy Does Not Improve Survival in cT2N0M0 Gastric Adenocarcinoma Patients: A Multicenter Propensity Score Analysis
Source: Ann Surg Oncol. 2024 May 18;31(8):5273–82. doi: 10.1245/s10434-024-15418-2 (PMC11236876; doi:10.1245/s10434-024-15418-2)
Supplement: Supplementary file 3 — Supplementary file3 (DOCX 20 KB) [file 10434_2024_15418_MOESM3_ESM.docx]

**Online Resource 3.** Univariate and multivariable Cox regression analysis for disease-free survival at five years, after inverse probability of treatment weighting.

| **Variable** | **Unadjusted**  **HR** | **95%CI** | **P Value** |  | **Adjusted**  **HR** | **95%CI** | **P Value** |
| --- | --- | --- | --- | --- | --- | --- | --- |
| Male sex | 1.67 | 0.82; 3.37 | 0.16 |  |  |  |  |
| Age (years) | 0.96 | 0.95; 0.98 | **<0.001** |  | 0.98 | 0.96; 1.00 | **0.02** |
| WHO performance status |  |  |  |  |  |  |  |
| 0 | 1.00 |  |  |  |  |  |  |
| 1 | 1.57 | 0.82; 3.00 | 0.17 |  |  |  |  |
| 2 | 0.66 | 0.13; 3.38 | 0.62 |  |  |  |  |
| 3 | - | - | NA |  |  |  |  |
| Smoking | 1.27 | 0.79; 2.04 | 0.32 |  |  |  |  |
| Alcohol consumption | 0.51 | 0.20; 1.32 | 0.17 |  |  |  |  |
| Tumor location |  |  |  |  |  |  |  |
| Proximal | 1.00 |  |  |  |  |  |  |
| Body | - | - | NA |  |  |  |  |
| Distal | 0.55 | 0.25; 1.23 | 0.15 |  |  |  |  |
| Diffuse | 1.40 | 0.21; 9.50 | 0.73 |  |  |  |  |
| pT stage |  |  |  |  |  |  |  |
| pT0 - pT1 - pT2 | 1.00 |  |  |  | 1.00 |  |  |
| pT3 - pT4 | 7.76 | 3.74; 16.12 | **<0.001** |  | 5.99 | 2.41; 14.86 | **<0.001** |
| pN stage |  |  |  |  |  |  |  |
| pN0 | 1.00 |  |  |  | 1.00 |  |  |
| pN1 | 2.81 | 1.18; 6.70 | **0.02** |  | 1.82 | 0.83; 4.02 | 0.14 |
| pN2 | 1.54 | 0.64; 3.72 | 0.34 |  | 1.61 | 0.48; 5.45 | 0.44 |
| pN3 | 1.65 | 0.30; 8.96 | 0.56 |  | 0.97 | 0.18; 5.16 | 0.97 |
| Dindo-Clavien grade |  |  |  |  |  |  |  |
| I - II - IIIa | 1.00 |  |  |  |  |  |  |
| IIIb - IVa - IVb | 1.97 | 0.80; 4.88 | 0.14 |  |  |  |  |
| Neoadjuvant chemotherapy | 1.02 | 0.50; 2.10 | 0.96 |  | 1.17 | 0.66; 2.08 | 0.59 |
| Poorly cohesive histology | 2.05 | 0.98; 4.28 | 0.06 |  | 2.00 | 1.13; 3.55 | **0.02** |
| Resection margins |  |  |  |  |  |  |  |
| R0 | 1.00 |  |  |  | 1.00 |  |  |
| R1 | 4.88 | 1.66; 14.31 | **0.004** |  | 1.89 | 0.75; 4.79 | 0.18 |
| Number of retrieved LN |  |  |  |  |  |  |  |
| <15 | 1.00 |  |  |  | 1.00 |  |  |
| ≥15 | 0.36 | 0.17; 0.78 | **0.009** |  | 0.31 | 0.15; 0.65 | **0.002** |
| *CI: confidence interval; HR: hazard ratio; LN lymph nodes; NA: not applicable; WHO: world health organization.* | | | | | | | |
